# Antibiotics and priority effects on colonizing resistant *Escherichia coli* strain assemblages in a community setting

**DOI:** 10.1371/journal.pone.0352163

**Published:** 2026-06-24

**Authors:** Eric Ng’eno

**Affiliations:** Biodiversity Institute, University of Kansas, Lawrence, Kansas, United States of America; Government College University Faisalabad, PAKISTAN

## Abstract

Colonizing antibiotic resistant bacteria often exists within functionally structured microbial communities, where occurrence is influenced by dependencies essential to stability and function. Considering these interactions could improve understanding of factors influencing diversity of colonizing resistant bacteria within human community settings. Using a framework that considered biotic interactions, we reexamined factors associated with assemblage of colonizing antibiotic resistant *Escherichia coli* strains in a densely populated urban informal settlement. We identified antibiotic selection, colonization legacy, positive feedbacks and dispersal as key assembly processes. Antibiotics and colonization legacy exerted strain-specific effects while dispersal factors influenced all strains rather uniformly. Specifically, areas frequently exposed to bactericidal antibiotics were dominated by locally established resistant strains, whereas areas frequently exposed to bacteriostatic antibiotics had increased presence of less-established strains. Assemblage configuration was influenced by relative abundance of strains initially present in and around the disturbed areas. Hygiene practices reinforced local strains consistently while weather variables had variable effects: high monthly average minimum temperatures increased abundance of nearly all strains, whereas increases in monthly average maximum temperature, humidity, and rainfall uniformly decreased strain abundance. Overall, we show that survival selection, driven by selective forces, and dispersal selection, shaped by colonization potential, jointly influenced strains recruitment and assembly, suggesting antibiotics and priority effects as factors contributing to resistant *E. coli* assemblages in this setting. Together these findings underscore need for context-specific antibiotic stewardship and hygiene interventions.

## Introduction

Carriage of bacteria resistant to antibiotics in community settings is influenced by complex suites of interacting risk factors that can be grouped broadly into those related to antimicrobial exposure, bacteria dissemination, and biotic interactions of different species and differentiated populations of the same species (henceforth referred to as ‘strains’) [1–4]. Human movement, sanitation, and hygiene factors facilitate broad bacterial dissemination [5–8]; contrasting levels of antibiotic exposure across geography over extended time periods influence divergent distributions in resistant bacteria strains occurrence [9]; while biotic interactions structure assemblages and potentially refine resistant strains distribution patterns [10]. Variation in community assemblages also may arise by ecological drift: random changes in the frequencies of strains at a location owing to dispersal, environmental, and demographic stochasticity [11–13].

Roles of antimicrobial exposure and bacterial dispersal (considered primary filtering forces) in shaping distribution of colonizing resistant bacteria in community settings, have been examined [6,14,15]. However, understanding of the role of biotic interactions remains incipient. Such forces, most apparent where resistant bacteria are already common, are challenging to measure [16–18]. Still, the potential effects of these forces are nontrivial [19–21]. Strains interactions integral to a microbial community’s stability and function, can influence exposure-resistance carriage relationships at local scales (i.e., the area over which individual interact) such that the presence or absence, and abundance of a strain in principle depends more on these interactions than on the primary filtering forces [19,22–27]. Moreover, biotic interactions can also confound dispersal-carriage associations in correlative analyses. For example, although unimpeded dissemination of resistant bacteria into a locality may cause spatial clustering, similar spatial clustering can also arise from positive density-dependent feedbacks.

Studies aiming to understand factors associated with carriage of bacteria resistant to antibiotics in community settings could therefore benefit from analyses that consider antibiotic exposure, dispersal factors and biotic interactions all together. However, consideration of biotic interactions in analytical frameworks for resistant bacteria carriage has lagged, likely due to data limitations, complexities and dynamisms of biotic interactions, and a lack of suitable analytical models [21,28]. Nevertheless, in highly connected settings, where strains can be considered to be at a greater degree of distributional equilibrium, such information may be captured by spatial variations of resistant strains abundances across a metacommunity [12,26–28]. This biotic context information can then be used in correlative, multi‑strain, analysis frameworks along with other environmental and dispersal covariates [29].

Multiple lines of evidence can serve as indicators for biotic interactions among colonizing antibiotic resistant bacteria within human community settings. Biotic interactions may be inferred when relative baseline densities of specific *Escherichia coli* strains predicts local assemblage structures, even after accounting for exposure, dispersal, and spatially structured random factors [22]. Presence of alternative stable states, reflected in bimodal distributions of colonizing resistant strains, may also signal underlying biotic processes, particularly when they emerge despite heterogeneous distribution of selective environments and unrestricted bacterial dispersal: limited dispersal can also result in bimodal distribution [29–33]. In this study, we tested for these indicators, and using a framework that implicitly considered the biotic environment, reexamined factors contributing to assemblage of local suites of resistant *E. coli* strains in a densely populated urban informal settlement [34,35].

## Methods

### Study site

We used data from a population-based infectious diseases surveillance (PBIDS) platform and from previous studies implemented on the same platform [9,36]. The surveillance is operated by the Kenya Medical Research Institute, in collaboration with the U.S. Centers for Disease Prevention and Control. The study area covers ~0.4 km^2^, and is characterized by inadequate sanitation, high infectious disease burden, high antibiotic use, and high prevalence of antibiotic-resistant *E. coli* colonization [37–39].

### Household and clinic surveillance

The surveillance data collection methods have been described [37]. Briefly, ~ 26,000 residents of all ages were followed each year through household- and clinic-based surveillance. Individuals had to have resided in the study area for a minimum of 4 months or be born to a PBIDS participant to be eligible to participate in the surveillance. During regular household surveys, community interviewers documented self- and proxy-reported demographic information (births, deaths, in-migrations, out-migrations) and data on recent illnesses and healthcare seeking. Demographic data were used to estimate participants’ duration of involvement in the study. PBIDS participants received medical care for acute infectious illness at no charge at a centrally located surveillance clinic (Tabitha Clinic). Based on household surveillance data, about 70% of the participants sought care at this clinic. Standardized clinical data, including information on filled antibiotic prescriptions, were collected at the clinic for participants meeting standardized case definitions [37].

### Proximity raster data layers

The preparation of raster data layers summarizing the spatial context of localities across the study area has been described in detail [9]. In brief, however, to generate layers estimating average distance (in meters) from each locality to the features assessed (drinking water sources, public toilets, dump sites, surface wastewater drainage networks and rivers, schools, clinics, and pharmacies), we used geographic point data for all the features within and immediately surrounding the study area, and polyline data for drainage networks and rivers. We then summarized the distance from all sites in the study area (i.e., 2m grid cells) to each of these features using the raster proximity analysis tool in QGIS (version 3.22.1).

### Antibiotic exposure raster data layers

To generate antibiotic exposure raster layers, we extracted data on prescriptions filled for participants presenting with acute infections at the surveillance clinic. We used filled prescriptions data for the period 1 January 2010 and 31 August 2015, for individuals whose household GPS coordinates were available at the time of the clinic visit. Antibiotic-related data extracted from the prescriptions included name, dose, daily units for consumption, and days of use. The data were extracted on 12 February 2020.

We estimated defined daily dosage (DDD) for each PBIDS household following World Health Organization (WHO) DDD recommendations [40]. For each antibiotic, we estimated the total dosage as a product of antibiotic unit strength in grams, number of daily prescribed units, and number of prescribed days of use. For individuals that received several prescriptions fills during the study period, we estimated dosage for each time point and added them together. We then estimated DDD by dividing the estimated dosage with the WHO-Anatomical Therapeutic Chemical (ATC)-assigned DDD index unit for the specific antibiotic (WHO, version 2022) [41].

To obtain household-level DDDs we summed DDDs for all individuals in a household. We divided household DDD by the total days of observation of the household to generate person-time-of-observation normalized household exposure estimates. We calculated total days of household participation in PBIDS as the sum of days of participation of all PBIDS members of the household during the period January 2010 to August 2015. Households that did not have any antibiotic exposure during the five-year period were considered to have zero exposure.

We extended the risk estimates across space by interpolating among all PBIDS households’ exposure point data using the cubic spline method in QGIS (version 3.22.1) [42]. We expressed exposure estimates in the form of DDD per 1000 inhabitant-days of observation (IDO).

### Background density raster data layers

These layers estimated baseline relative abundance of specific resistant strains at each locality in the study area. The estimates were used to approximate biotic conditions in different localities at the start of survey [32]. While these estimates ideally would be derived from environmental sampling, in this study we used as proxy, proportions of specific resistant strains derived from stool samples of individuals enrolled in the study. A prior study in the setting showed that loads of resistant *E. coli* strains in stool samples were generally consistent with those from wastewater samples collected in same locality [43]. Only samples collected during the first round of survey were used. We excluded households that began contributing samples in the second round (≥14 days after survey initiation). Estimates of specific resistant *E. coli* strains abundance in each locality sampled were calculated by dividing the total number of the resistant colonies identified into number of colonies tested for resistance in the locality. We extended the density estimates between the sampled localities by interpolating among localities estimates using the cubic spline method in QGIS (version 3.22.1) [42]. Estimates were restricted within a 0–1 range.

### Weather data

We used monthly average data on humidity, precipitation, maximum and minimum temperature obtained from the Kenya Meteorological Department. These data were sourced from two stations: Dagoretti Weather Station (1° 18’ 1.8612” S, 36° 45’ 38.412” E), located ~2 km northwest of the study area, and Wilson Airport Weather Station (1° 19’ 14.0592” S, 36° 48’ 55.6056” E), located ~4.3 km southeast of the study area. Daily observations for each variable were used to calculate monthly averages for each station. These values were then averaged across the two stations for representative estimates of the study area which was placed between the two stations. The weather data covered the period September 2015 to January 2016. Given the small size of the study area, weather was assumed to vary only temporally, and local microclimatic variation was neglected.

### Antibiotic resistance colonization

We used data on resistant *E. coli* colonization in asymptomatic individuals from a previous longitudinal study conducted in the same PBIDS population. Sample collection and processing methods have been described elsewhere [36]. Briefly, however, 200 households were selected randomly from among households with at least one adult (≥18 years old) and one child (≤5 years old) from each housing block across the study area. The approach aimed at ensuring demographic and spatial representation. Households selected were distributed across 195 unique locations, as defined by their geocoordinates. Households shared the same geocoordinates if they shared a roof and entrance point.

Households were sampled approximately every two weeks between September 2015 and January 2016. Two stool samples were collected (from an adult, and from a child when available) per household. A gram of stool from each sample was emulsified in phosphate-buffered saline, serially diluted, and then plated on MacConkey agar using sterile glass beads. Twelve presumptive *E. coli* (24 for two samples per household) were selected randomly from each plate, and sub-cultured on nutrient broth in a 96-well plate.

A 96‐well pin replicator was then used to transfer isolates onto 150 mm MacConkey agar plates, each with one of six antibiotics (32 μg/mL ampicillin; 8 μg/mL ceftazidime; 32 μg/mL chloramphenicol; 4 μg/mL ciprofloxacin; 64 μg/mL sulfamethoxazole; and 16 μg/ml trimethoprim, all from Sigma, St. Louis, MO). A plate without antibiotics was included to confirm cell viability. Isolates were scored as resistant (complete growth) or susceptible (no growth or partial growth). To estimate the proportion of resistant *E. coli* per gram of stool in an individual, the number of identified resistant colonies was divided by the total number of colonies tested for resistance from that individual.

### Data organization

For model fitting, we compiled a final dataset of resistant *E. coli* colonization that excluded data points from the first round of survey (31 August 2015–13 September 2015) used for background density estimation. Since we set out to evaluate association between predictor variables and occurrence of colonizing antibiotic resistant *E. coli* strain assemblages (rather than their relative abundance), we transformed the resistance proportions into presence-absence (1 if >0, and otherwise 0). Additionally, we transformed all environmental predictor raster layers to same spatial resolution (4 x 4 m) and spatial extent (1516 x 476 m) using raster aggregation (average of values) procedures; one antibiotic exposure raster layer (amoxycillin layer) was used as a reference to which pixels of all other raster layers were matched at each resolution using nearest neighbor-aggregation method in the raster package (version 3.6–26) in R [44,45].

For each set of coordinates of households tested for antibiotic-resistant *E. coli* colonization, we identified the raster cell containing the coordinate and assigned estimated values for the different risk parameters explored. We also added weather data, household-level hygiene practices, as well as the age of individuals tested at each household. We standardize each continuous predictor variable and binarized (0,1) categorical and age (0 if adult, 1 if child) variables. To account for potential quadratic relationships, we also generated quadratic forms of antibiotic exposure and weather variables.

### Descriptive analysis

We summarized proportions of resistant *E. coli* over all antibiotics tested for resistance. We considered colonization with susceptible *E. coli* where all colonies were sensitive to all the antibiotics tested. Because of the compositional nature of the resistance data, we used the framework Sparse Correlations for Compositional data (SparCC) [46,47] to infer correlations among the resistant *E. coli* strains observed. The analysis was performed using R package *SpiecEasi* [48].

### Bimodality testing

To assess potential bimodality of patterns in the carriage of each resistant *E. coli* strain, we plotted frequency distribution of the strain’s relative abundances across sampling sites. We then compared the observed distribution with distribution expected by chance. Null relative abundance distributions were generated by randomly extracting the absolute abundance values of a focal strain and those of non-focal strain from across households sampled, and then calculating their relative abundance [49,50]. The sample size of the subsamples matched that of the total sample tested for resistance. For each strain, we repeated the process 1000 times and estimated median frequency and 95% confidence interval of number of sites with the different relative abundances. We used 0.1 as the relative abundance bin width.

We also compared Spearman’s correlation coefficients calculated from the observed data with those expected by chance (obtained through random subsampling in the null model simulations). We hypothesized that, in a bistable distribution, strains resistant and susceptible to specific antibiotics will be more anticorrelated than would be expected by chance.

### Statistical analysis

Following Bauer et al. [51], we used the jSDM framework via the R package *Hierarchical Modeling of Species Communities* (HMSC) to assess the influence of our predictor variables on the diversity of resistant *E. coli* strains in the area [52,53]. To account for multicollinearity, we calculated variance inflation factor (VIF) for each strain and retained variable sets with VIF values <10 [54]. These variables were added into the jSDM as fixed effects. We also added site and spatial random effect variables to account for repeat sampling and residual spatial autocorrelation, respectively. We used enzyme-mediated antibiotic inactivation resistance mechanism as a grouping trait given its potential use in population- and community-wide protection through antibiotic inactivation and community signaling [4,27,55,56]. Except for the susceptible strain and those resistant to ciprofloxacin, streptomycin (resistance typically mediated by point mutations [57,58]), and tetracycline, all of the other six strains were considered to rely primarily on enzymatic resistance mechanisms. [59,60].

We used a probit distribution owing to the binary nature of our response variables. We then sampled the posterior distribution with default prior distributions using three Markov chain Monte Carlo (MCMC) chains, based on the Gibbs sampling approach implemented in HMSC [52]. Each chain ran for 300,000 iterations, with the first 50,000 as burn-in. The remaining iterations were thinned every 1000 iterations to yield 250 posterior samples per chain and 750 samples in total. We assessed model convergence by inspecting posterior trace plots and by evaluating potential scale reduction factors (psrf) of the model parameters [61]. We examined the explanatory power using strain-specific coefficients of discrimination (Tjur’s *R*^*2*^) [62] and evaluated model classification performance using the area under the receiver operating characteristic curve (AUC). Models were evaluated for their ability to predict the occurrence distribution of all strains simultaneously as well as each strain individually. Associations between strains and the covariates were assessed using Beta parameter. An effect was considered statistically supported where the posterior support exceeded 0.95.

To measure relative contribution of the predictors to the strain occurrences, we partitioned the total variance as measured by Tjur’s *R*^*2*^ for both fixed and random effects. Relative proportions of variance explained were estimated both by grouped categories and by individual risk factors (Table 1).

**Table 1:** Variables and their corresponding categories used in this analysis.

Variable category	Variables
Dispersal related factors	Water.available.source,Water.source.safe, Treat.water,Boil.water,Chemical.water.treat, Toilet.location,Toilet.cleaned, Toilet.cleaning.freq [> 6], HW.station.in.premise, HW.station.out.premise, No.designate.HWplace, Child.play.away.home,Child.eatsoil, Share.toilet [1–5],Water.treatment.always, Proximity.to.chemists, Proximity.to.rivers.and.ditches, Proximity.to.dumpsites,Proximity.to.schools, Proximity.to.toilets,Proximity.to.waterpoints, Population.density
Selective factors	Amoxycillin,Ampiclox,Azithromycin, Benzathine.penicillin,Ceftriaxone, Cefuroxime,Cephalexin,Ciprofloxacin, Cloxacilin,Doxycycline,Erythromycin, Flucloxacillin,Gentamycin,Metronidazole, Nitrofurantoin,Norfloxacin, Phenoxymethylpenicillin, Trimethoprim.sulfamethoxazole,Amoxycillin.2, Ampiclox.2,Azithromycin.2, Benzathine.penicillin.2,Ceftriaxone.2, Cephalexin.2,Ciprofloxacin.2,Cloxacilin.2, Doxycycline.2,Flucloxacillin.2,Gentamycin.2, Metronidazole.2,Nitrofurantoin.2, Norfloxacin.2,Phenoxymethylpenicillin.2, Trimethoprim.sulfamethoxazole.2
Baseline strains density	Ampicillin.density,Ceftazidim.density, Chloramphenical.density, Ciproflaxicin.density,Kanamycin.density, Streptomycin.density, Sulfamethoxazole.density, Tetracycline.density,Trimethoprim.density, Susceptibles.density
Weather	Ave.Humidity,Ave.Maxtemp,Ave.Mintemp, Ave.Monthlyrainfal
Age	Age

HW – Handwashing, Ave – Average

We also assessed residual correlation patterns in models incorporating and excluding background resistant strains abundance estimates, to separate effects of the biotic environment. Specifically, we assessed the extent to which observed correlation among the strains could be explained by initial distribution patterns of strains’ abundance in the area. The models were fitted as described and only varied in consideration of the baseline strains abundance estimates. Since interaction effects often are density-dependent, and thus apparent where resistant bacteria are already common, we restricted our definition of sites colonized to those where at least 25% of total tested isolates were resistant to an antibiotic.

### Ethics statement

Ethical approvals for the studies whose data were used in this analysis were obtained from the KEMRI Scientific and Ethics Review Committee (SSC protocol numbers 3928, 2998, 1899 & 2761) and the US CDC Institutional Review Board (protocol numbers 4566 & 6775). Participation in each study was voluntary and participants could decline participation at any time. For PBIDS, written informed consent was obtained from heads of households for their household members to participate, with individual members retaining the right to decline participation. For the carriage study, additional written informed consent was obtained from household heads and participating adults prior to study enrollment. For children aged < 7 years, parental consent was obtained, by the parent or guardian, whereas for children aged 7–17 years, parental consent and a written assent from the child, were required. All data analyzed were anonymized to protect participant confidentiality.

## Results

Between 1 September 2015 and 22 January 2016, 2320 stool samples were collected from households across 195 distinct locations within the study area. Of these, 1212 (52.2%) samples were from children under five years old and 1108 (47.8%) were from adults 18 years old and above. Of the total, 321 (13.8%) samples were used for background resistance estimation, and 1949 (97.5%) of the remaining 1999 samples could be linked to all predictor variables and were included in our statistical analysis.

**Fig 1:**
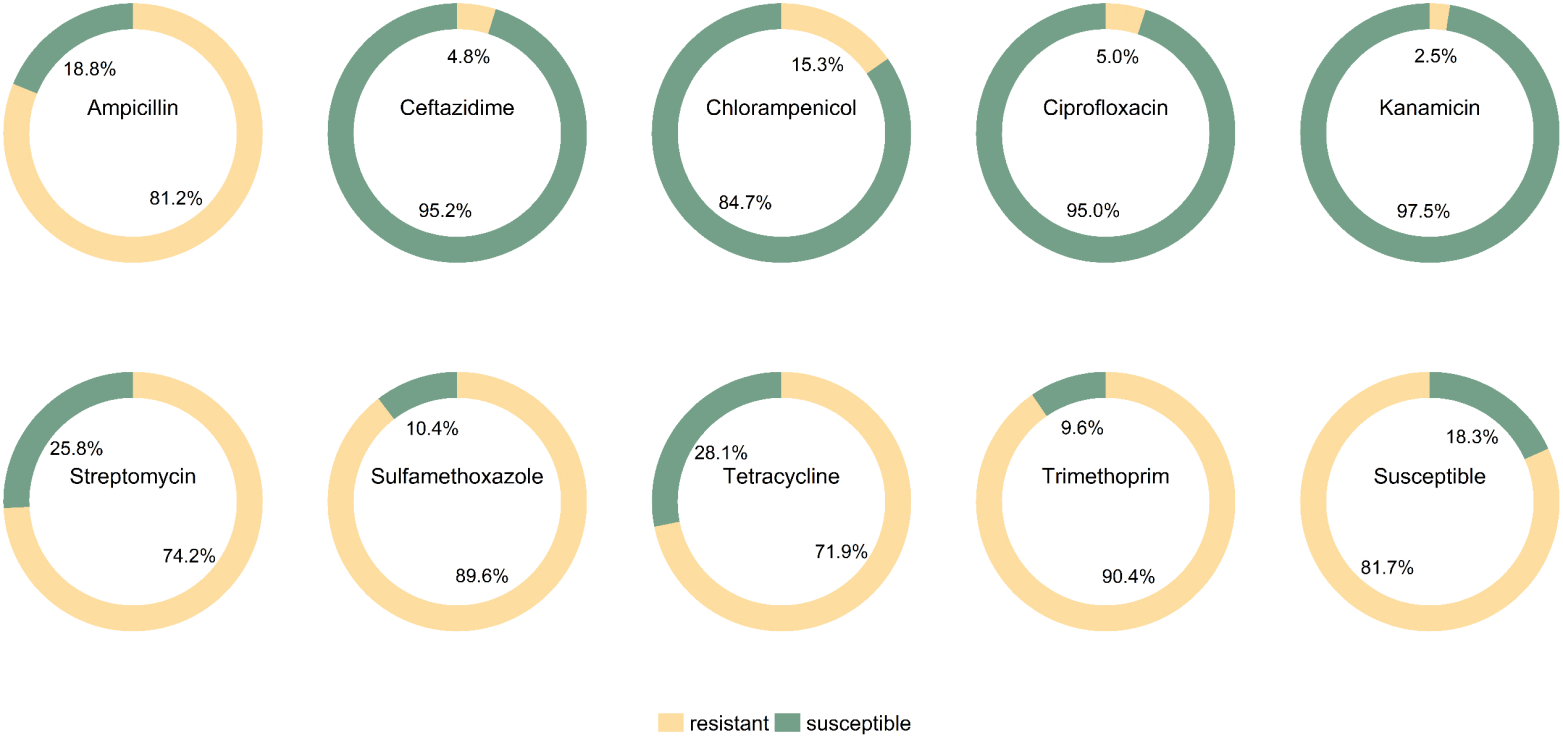
Antibiotic susceptibility of colonizing *Escherichia coli.* Proportion of samples colonized with specific antibiotic-resistant *Escherichia coli* and samples colonized with *Escherichia coli* susceptible to all tested antibiotics. Proportions estimated with total stool samples collected (n = 2320).

Overall, the proportion of stool samples colonized with resistant *E. coli* strains ranged from 57 of 2320 (2.5%) for kanamycin to 2098 of 2320 (90.4%) for trimethoprim. Stool samples colonized with susceptible *E. coli* were 425 (18.3%) (Fig 1). When we examined the distribution of resistant *E. coli* colonization by age groups, patterns similar to those in the overall trend were observed (S1 Fig).

**Fig 2:**
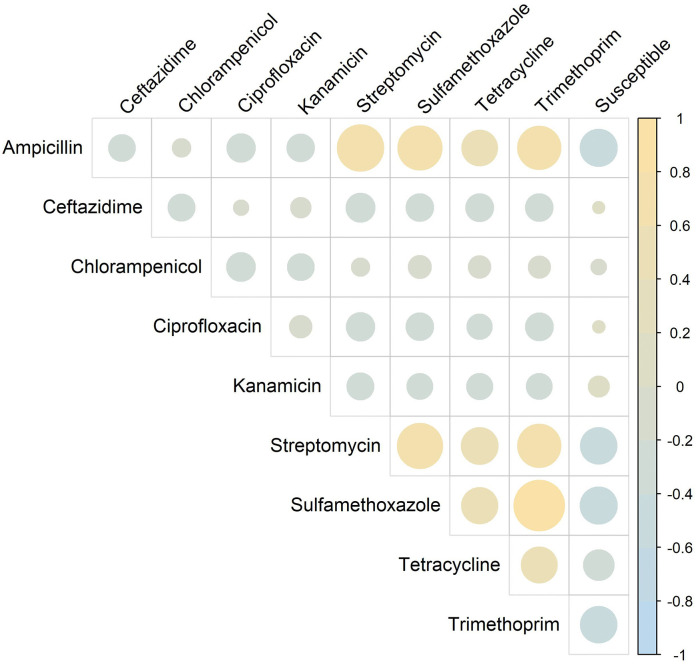
Correlation among *Escherichia coli* strains. Correlations inferred using sparse correlations for compositional data method. Yellow color indicates positive correlation while blue negative correlation among the strains. The bubble size represents the strength of the correlation. Correlation estimated with total stool samples collected (n = 2320).

We observed positive correlation among strains resistant to ampicillin, streptomycin, sulfamethoxazole, tetracycline, and trimethoprim (which we describe as the common resistant strains) (Fig 2), and a negative correlation between these strains and the other strains, especially susceptible strains. The susceptible strain did not covary with strains resistant to ceftazidime, ciprofloxacin, chloramphenicol or kanamycin (which we describe as the rare resistant strains).

**Fig 3:**
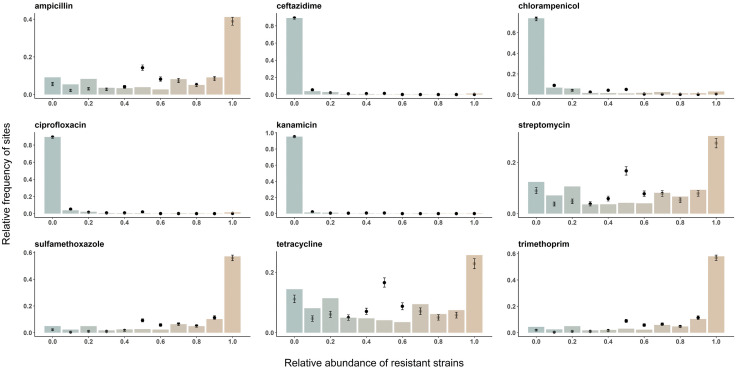
Gradient-colored histogram showing frequency distribution of colonizing resistant *Escherichia coli* relative abundances by site (green, ~ 100% susceptible; brown, ~ 100% resistant). The black dots represent the median, while the error bars indicate the 2.5th and 97.5th quantiles of the distribution obtained through random subsampling (*n =* 1000) in the null model simulations. Proportions were estimated using total number of household samples tested (n = 2320).

Assessment of the frequency distribution of relative abundances of specific resistant strains by locality showed bimodal patterns in carriage of *E. coli* resistant to ampicillin, streptomycin, sulfamethoxazole, tetracycline, and trimethoprim. Sites were either dominated by strains resistant to these antibiotics or susceptible to them, with those colonized by both types appearing less commonly than would have been expected by chance (Fig 3). Similarly, strains resistant and susceptible to specific antibiotics were more anticorrelated than expected by chance in the Spearman correlation analysis (S2 Fig).

**Fig 4:**
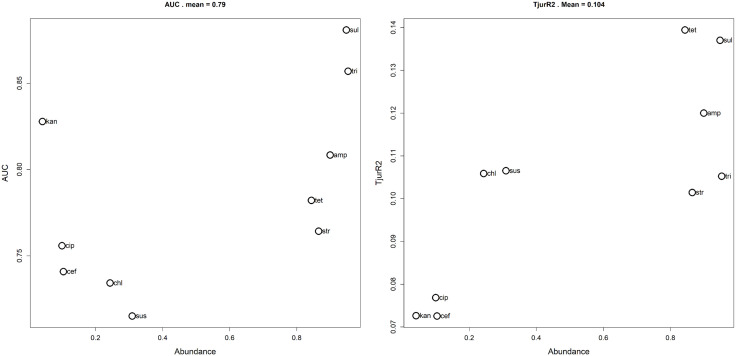
Model classification and explanatory performance as measured by area under the receiver operating characteristic curve AUC and Tjur *R*². Each point per plot represents a resistant strain while corresponding x-axis shows the strain’s abundance estimate. Left panel represents AUC estimates while right panel estimates Tjur *R*^*2*^ for each strain. Amp, ampicillin; cip, ciprofloxacin; cef, ceftazidime; chl, chloramphenicol; kan, kanamycin; sul, sulfamethoxazole; sus, susceptible; str, streptomycin; tet, tetracycline; tri, trimethoprim.

In total, 88 variables were generated, of which 72 were used to fit the jSDM model, after exclusion of variables with VIF values ≥10 (S1 Table). Assessment of potential scale reduction factors showed convergence in model parameters across the MCMC chains (psrf < 1.1) (S3 Fig). The mean AUC of the models was 0.79, and they explained 10% of variance in the resistant *E. coli* strain diversity in the area (Fig 4). Higher AUCs and Tjur’s *R*^*2*^ were observed for strains that were present in higher abundance.

**Fig 5:**
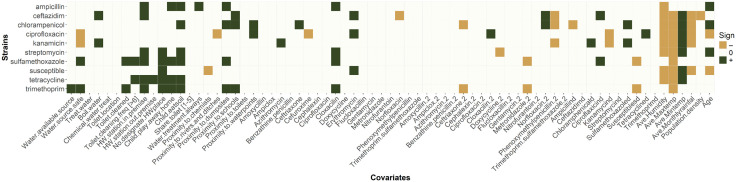
Correlates of *Escherichia coli* strains occurrence. Correlations among dispersal-related factors; selection factors; baseline strains density; weather; age; and the occurrence of resistant *Escherichia coli* strains. Quadratic terms for antibiotic exposure variables are denoted by adding “.2” to the antibiotic name. Susceptible strains are sensitive to all antibiotics tested. Green indicates a positive effect (beta parameter), while orange indicates a negative effect. White indicates no statistically significant effect. Effects with posterior support greater than 0.95 considered statistically significant.

Analysis of factors associated with occurrence of resistant *E. coli* strains in the area showed significant effects of hygiene- and sanitation-related factors. Hygiene practices such as frequent toilet cleaning, regular hand washing, sharing toilets, along with sanitation factors including children’s direct exposure to the environment, living in areas away from waste dump sites and schools, were associated with increased colonization by the common resistant strains (Fig 5).

Associations were also observed for specific antibiotics. For instance, individuals residing in areas with increased exposure to cloxacillin were associated with increased risk of carrying the common resistant strains, whereas those residing in areas with increased erythromycin exposure had increased carriage risk for ceftazidime- and ciprofloxacin-resistant strains, and *E. coli* susceptible to all antibiotics. Quadratic forms of antibiotics were generally associated with negative responses, indicating presence of intermediate optimum in the antibiotics selective environment.

The underlying relative abundance of specific resistant strains influenced the occurrence of other strains. For example, areas that had high ampicillin-resistant strain abundance had reduced frequency of chloramphenicol-resistant strains. Similarly, presence of strains fully susceptible to all antibiotics tested was inversely associated with occurrence of strains resistant to sulfamethoxazole and trimethoprim.

Seasonal increase in average monthly humidity, maximum temperature and rainfall, were associated with decreased frequency of most strains examined. In contrast, higher average minimum temperature was associated with increased frequency of nearly all strains assessed. Children were more frequently colonized by the common resistant strains in the area, with the fully susceptible strain identified among them only infrequently.

**Fig 6:**
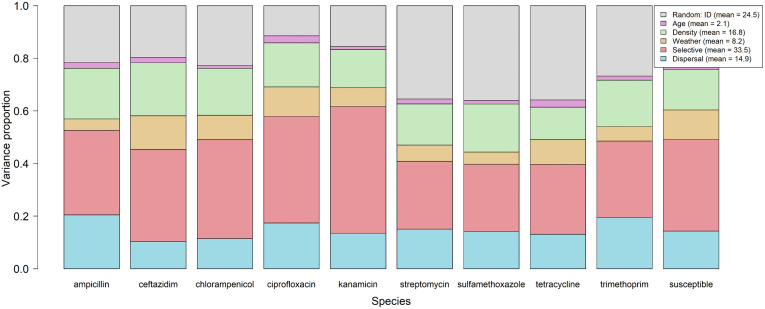
Proportion of total variance explained by predictor categories. The relative contribution of each predictor group to the total variance explained (see Fig 4, right panel) is shown for individual strains. Predictor groups include spatially structured random effects (random ID), age, baseline strain abundance (density), weather‑related factors (weather), selective factors related to antibiotic exposure (antibiotic) and dispersal‑related factors (dispersal). Bar colors indicate the proportion of variance explained by each predictor category for each strain.

When we partitioned the variance explained, across strains, selective factors explained most of observed variance in strain assemblages (33.5%) followed by spatial random effects (24.5%), baseline strain densities (16.8%) and dispersal-related factors (14.9%) (Fig 6). These factors varied by strain type. For instance, spatial random effects explained more variance among the common resistant strains; in the relatively rare resistant strains, however, most variance was explained by selective factors.

**Fig 7:**
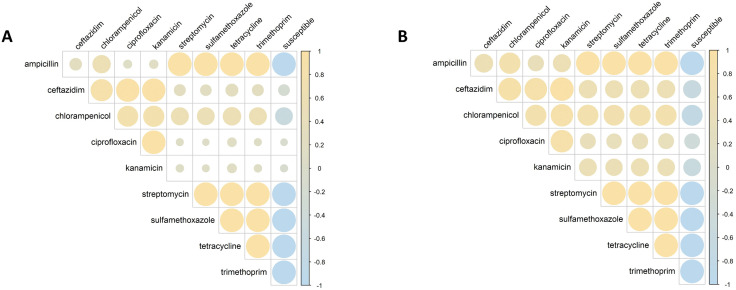
Residual joint species distribution model estimates among *Escherichia coli* strains. Panel (a) show residual correlation of models excluding baseline strain abundance estimates while panel **(b)**, show residual correlation of models accounting for baseline strain abundance. The bubble size represents the strength of the correlation. Yellow color indicates positive correlation while blue negative correlation.

Residual correlations from models excluding background strain densities revealed two distinct groups: one comprising common resistant strains that were positively correlated with each other but negatively correlated with the susceptible strain, and another consisting of rare resistant strains that also showed positive intercorrelation but no variation with the susceptible strain (Fig 7a). In contrast, residual correlations from models that accounted for background strain densities showed that nearly all resistant strains were positively correlated and negatively correlated with the susceptible strain (Fig 7b).

## Discussion

Understanding the risk factors for resistant *E. coli* colonization is important for designing and implementing antibiotic resistance control interventions. Using a framework that incorporated biological interactions, we set out to determine factors, that, shape assemblage of antibiotic-resistant *E. coli* strains in a densely populated setting, where resistant bacteria carriage is prevalent [36,63]. We found antibiotic selection, colonization legacy, positive density-dependent feedbacks, and dispersal as key measured assembly processes shaping strains assemblages in the area. Different antibiotics and starting local strain abundances each influenced occurrence of distinct configuration of resistant strains. In contrast, hygiene and weather-related factors were more general and did not affect specific strain types but rather exhibited a more uniform influence, simultaneously increasing or decreasing occurrence of strains. The influence was local (i.e., on strain sets in specific areas of influence), for hygiene factors that varied geographically, and global (all strains identified) for weather factors which varied only temporally. Hygiene practices were unexpectedly associated with increased risk of resistant bacteria carriage, and children mainly carried the common resistant strains potentially owing to their increased exposure to environment [64].

Unlike other antibiotics with effects observed only on specific strains, cloxacillin and erythromycin, both commonly used in this setting [9], exerted consistently uniform effects across specific strains set. Areas with increased exposure to cloxacillin, a narrow-spectrum, bactericidal antibiotic, were predominantly colonized by the common and likely cloxacillin-tolerant strains, a pattern consistent with secondary succession [60,65–67]. Although cloxacillin is not generally active against Enterobacteriaceae, its disruption of other gut microbiota members likely confers these strains, already established in the local and regional pools, a competitive edge during recruitment and community assembly such that they can attract the community towards states in which they are dominant over other *E. coli* strains [12,68,69]; similar patterns could also arise owing to loss of an interacting strain or species key to an alternative configuration [4,70]. On the contrary, rare resistant and susceptible *E. coli* strains were more frequently observed in areas with recurrent erythromycin exposure. This assembly configuration could be attributed to erythromycin’s broad-spectrum bacteriostatic activity, which likely induces population bottlenecks and subsequent community drift, potentially reordering competitive dominance and assembly reconfigurations [71]. Additionally, its antagonistic interactions with other antibiotics likely creates ecological refugia where diverse strains could coexist [72–74].

The strains formed two distinct groups despite heterogeneous distributions of selective environments and an unrestricted bacteria dispersal potential in the area. Group membership was associated with local underlying abundances of specific resistant strains, some of which individually exhibited bimodal distribution. Together, these findings suggest effect of colonization legacy along with positive density-dependent feedback mechanisms in assembling of communities in which the resistant strains occur [12,75]. It also suggests existence of at least two alternative, potentially functional, stable states in the colonizing microbial community in this setting. Existence of alternative stable states in gut microbiota has been described [69,76]. Following recolonization of disturbed environments, different resistant strains can modify their environments to reinforce their dominance through secretion of inhibitory molecules, modification of local pH, and inflammation processes that modify oxygen availability at a site [12,68,77].

Our analysis of multiple strains concurrently showed that responses to hygiene- and weather-related factors were generally not strain specific, and tended to be uniform across all strain types present in a given setting; although this pattern may vary where different species are assessed. This finding indicates that these factors are not selective in and by themselves but instead create environments equally permissive for dissemination of strains present in a setting, a passive role that has been previously suggested [78]. For example, frequent hand washing likely disrupts colonization resistance opening a site for colonization by strains present in the broader area. On the other hand, increased minimum temperatures can increase human activity, facilitating movement of bacteria, while high rainfall and temperature likely reduce human interactions, and washout bacteria present in the environment, all which reduce opportunities for bacteria to spread. Increased bacteria replication and dispersal owing to increasing minimum temperature has also been suggested [79].

Further, we showed that, in this densely populated setting, where resistant bacteria carriage is prevalent and bacteria disperse freely, household-level hygiene practices increased risk of resistant bacteria carriage, a finding consistent with a previous study in the area which assessed individual-level risk factors [36]. Aspenberg *et al* [80] found that by limiting mixing of bacterial strains, hygiene measures interact with antibiotic exposure to dampen expansion of resistant bacteria strains in a population. Here, we clarify that this relationship is likely context dependent: in settings where resistant strains dominate, hygiene measures intended to reduce bacterial mixing may unexpectedly interact with antibiotics to reinforce the resistant strains and block expansion of rare susceptible variants assuming the equal probability of dispersal. This idea that limiting bacterial mixing promotes persistence of locally adapted strains is further supported in our observation that areas distant from those with potentially high strain diversity showed increased occurrence of common resistant strains.

The residual correlation from the model, which accounted for all measured variables and spatially structured random effects, revealed a clustering of nearly all resistant *E. coli* strains that was mostly anticorrelated to the susceptible strain. This pattern suggests the influence of additional, unmeasured factors that lack a spatial signature, given that our analysis already considered spatially structured unmeasured variables through the random effect variable [81]. We attribute this clustering, at least in part, to potential evolutionary differences between resistant and susceptible strains [82]. Specifically, the grouping likely reflects evolutionary relatedness in genetic mechanisms underlying global resistance traits such as efflux pumping and porin modulations among the resistant *E. coli* strains [83]. Variations in host resources and space availability which were not considered in this study, may also affect assemblage patterns. [67].

These findings are reported with several limitations and caveats regarding their interpretation. We only considered interactions among *E. coli* strains, yet multiple bacteria species colonize the gut such that they may interact with *E. coli* strains to influence assemblages observed [27]. We did not consider genomic data, which could clarify which resistant strains were colonizing the population. Some strains can carry multiple resistance genes [84], complicating discernment of whether the assemblages we observed were the result of interactions between different strains or fewer strains that carried multiple resistance genes. Further, the availability of genetic data could also have provided clarity regarding the residual correlation. A considerable portion of variance explained was attributed to spatially structured random factors, highlighting the need for further studies to identify these factors. A relatively small, highly interactive area was studied, which may limit clear delineation and likely stable distribution of assemblage patterns that might be evident across a broader area. Similarly, some strains were underrepresented which may hinder model’s ability to distinguish true habitat signals from background noise. These limitations along with the overall limited scope of the study make it difficult to apply our findings more broadly. Expanding geographic scope and ecological context in future work could help clarify these relationships.

In summary, we identified antibiotic, colonization legacy, positive feedbacks and dispersal as key long-term assembly processes shaping colonizing resistant *E. coli* strain diversity in this setting. We show that both survival selection (shaped by selective forces) and dispersal selection (largely influenced by invasion capability) shape recruitment, with strain assembly determined by the relative abundance and self-reinforcement of the strains initially present in and around a locality. Together this suggests that antibiotic exposure and priority effects interact to shape the diversity of colonizing resistant *E. coli* in a setting. These findings underscore the importance of context-specific approaches to antibiotic stewardship and hygiene interventions. Settings dominated by resistant types may benefit from promoting immigration and widespread dissemination of wild-type strains, coupled with antibiotics that promote drift, while limiting microbial dispersal through hygiene measures may be more effective in wild-type-strains, dominated areas.

## Supporting information

S1 Table Variables explored vs those selected for modeling. Variables not included in the final model are blank on the second column.
(DOCX)

S1 Fig Antibiotic susceptibility of colonizing *Escherichia coli* by age group. Proportion of samples colonized with specific antibiotic-resistant *Escherichia coli* and samples colonized with *E. coli* susceptible to all tested antibiotics by age group. Panel (a) shows proportions in samples collected from children under five years of age while panel (b) shows proportions among samples collected from adults eighteen years and above of age (n = 2320).
(TIF)

S2 Fig Spearman’s correlation coefficient comparison. Spearman’s rank correlation coefficient of specific resistant strains (orange line) compared to the distribution of coefficients obtained through random subsampling (n = 1000) in the null model (gray histogram). The coefficients were calculated using total number of household samples tested (n = 2320).
(TIF)

S3 Fig Model diagnostics. MCMC chain convergence diagnostics using potential scale reduction factors (psrf) of beta-parameters (strains-environment, mean point estimates (est.) = 1.004 [mean upper credible interval (CI) = 1.016]) and gamma parameters (trait-environment, mean point est. = 1.003 [mean upper CI = 1.02].
(TIF)
