# Supplementary material for: Antibiotics and priority effects on colonizing resistant Escherichia coli strain assemblages in a community setting
Source: PLoS One. 2026 Jun 24;21(6):e0352163. doi: 10.1371/journal.pone.0352163 (PMC13293415; doi:10.1371/journal.pone.0352163)
Supplement: S1 Table — Variables not included in the final model are blank on the second column. (DOCX) [file pone.0352163.s001.docx]

| All variables | Variables included for modelling |
| --- | --- |
| Chemical.water.treat | Chemical.water.treat |
| Age | Age |
| Amoxiclav |  |
| Amoxiclav.2 |  |
| Amoxycillin | Amoxycillin |
| Amoxycillin.2 | Amoxycillin.2 |
| Ampicillind | Ampicillind |
| Ampiclox | Ampiclox |
| Ampiclox.2 | Ampiclox.2 |
| No.designate.HWplace | No.designate.HWplace |
| Avg.Maxtemp | Avg.maxtemp |
| Avg.Mintemp | Avg.mintemp |
| Avg.Monthlyrainfall | Avg.monthlyrainfall |
| Avg.Humidity1200z | Avg.humidity1200z |
| Azithromycin | Azithromycin |
| Azithromycin.2 | Azithromycin.2 |
| Benzathine.Penicillin | Benzathine.penicillin |
| Benzathine.Penicillin.2 | Benzathine.penicillin.2 |
| Boilwater | Boilwater |
| Ceftazidimd | Ceftazidimd |
| Ceftriaxone | Ceftriaxone |
| Ceftriaxone.2 | Ceftriaxone.2 |
| Cefuroxime | Cefuroxime |
| Cefuroxime.2 |  |
| Cephalexin | Cephalexin |
| Cephalexin.2 | Cephalexin.2 |
| Child.play.away.home | Child.play.away.home |
| Chloramphenicald | Chloramphenicald |
| Ciproflaxicind | Ciproflaxicind |
| Ciprofloxacin | Ciprofloxacin |
| Ciprofloxacin.2 | Ciprofloxacin.2 |
| Cloxacilin | Cloxacilin |
| Cloxacilin.2 | Cloxacilin.2 |
| Doxycycline | Doxycycline |
| Doxycycline.2 | Doxycycline.2 |
| Child eatsoil | Child eatsoil |
| HW.station.in.premise | HW.station.in.premise |
| Erythromycin | Erythromycin |
| Erythromycin.2 |  |
| Flucloxacillin | Flucloxacillin |
| Flucloxacillin.2 | Flucloxacillin.2 |
| Gentamycin | Gentamycin |
| Gentamycin.2 | Gentamycin.2 |
| Humidity1200z.2 |  |
| Kanamycind | Kanamycind |
| Levofloxacin |  |
| Levofloxacin.2 |  |
| Avg.Maxtemp.2 |  |
| Metronidazole | Metronidazole |
| Metronidazole.2 | Metronidazole.2 |
| Avg.Mintemp.2 |  |
| Monthlyrainfall.2 |  |
| Nalidixic.Acid |  |
| Nalidixic.Acid.2 |  |
| Nitrofurantoin | Nitrofurantoin |
| Nitrofurantoin.2 | Nitrofurantoin.2 |
| Norfloxacin | Norfloxacin |
| Norfloxacin.2 | Norfloxacin.2 |
| Nowatertime |  |
| HW.station.out.premise | HW.station.out.premise |
| Phenoxymethylpenicillin | Phenoxymethylpenicillin |
| Phenoxymethylpenicillin.2 | Phenoxymethylpenicillin.2 |
| Population.density | Population.density |
| Proximity.to.chemists | Proximity.to.chemists |
| Proximity.to.dumpsites | Proximity.to.dumpsites |
| Proximity.to.rivers.and.ditches | Proximity.to.rivers.and.ditches |
| Proximity.to.schools | Proximity.to.schools |
| Proximity.to.toilets | Proximity.to.toilets |
| Proximity.to.waterpoints | Proximity.to.waterpoints |
| Share.toilet [1-5] | Share.toilet [1-5] |
| Share.toilet >5 |  |
| Sites | Sites |
| Streptomycind | Streptomycind |
| Sulfamethoxazoled | Sulfamethoxazoled |
| Susceptiblesd | Susceptiblesd |
| Tetracyclined | Tetracyclined |
| Toiletcleaned | Toiletcleaned |
| Toilet cleaning frequency | Toilet cleaning frequency |
| Toiletlocation | Toiletlocation |
| Treatwater | Treatwater |
| Trimethoprim.Sulfamethoxazole | Trimethoprim.sulfamethoxazole |
| Trimethoprim.Sulfamethoxazole.2 | Trimethoprim.sulfamethoxazole.2 |
| Trimethoprimd | Trimethoprimd |
| Water.source.safe | Water.source.safe |
| Water.available.source | Water.available.source |
| Water.treatment.always | Water.treatment.always |
| Water.treatment.once in a while |  |
| Water. Treatment.Not applicable |  |
