# Supplementary material for: Antibiotics and priority effects on colonizing resistant Escherichia coli strain assemblages in a community setting
Source: PLoS One. 2026 Jun 24;21(6):e0352163. doi: 10.1371/journal.pone.0352163 (PMC13293415; doi:10.1371/journal.pone.0352163)
Supplement: Attachment — Submitted filename: Response to Reviewers.docx [file pone.0352163.s006.docx]

**Response to Reviewers**

PONE-D-26-03469R1

Antibiotics and priority effects shape colonizing resistant *Escherichia coli* strain assemblages in a community setting

To the Editor:

I thank you for your time and consideration of this manuscript submitted to PLoS One for publication. I also thank the reviewers for their time and constructive comments.

I have revised the manuscript in response to comments and suggestions made. Please find the detailed responses in blue

Please advise if there will be anything else needed.

Sincerely,

Eric Ng'eno

**Journal Requirements:**

When submitting your revision, we need you to address these additional requirements.

1. Please ensure that your manuscript meets PLOS ONE's style requirements, including those for file naming. The PLOS ONE style templates can be found at

[https://journals.plos.org/plosone/s/file?id=wjVg/PLOSOne_formatting_sample_main_body.pdf](https://track.editorialmanager.com/CL0/https:%2F%2Fjournals.plos.org%2Fplosone%2Fs%2Ffile%3Fid=wjVg%2FPLOSOne_formatting_sample_main_body.pdf/1/010f019d1ea72099-7699703e-35be-4396-b571-8bcffeae5500-000000/gELfCnqjIyHgwTZEZfwT_mjJP2ZIilGkOmgUZ29yvWg=255) and [https://journals.plos.org/plosone/s/file?id=ba62/PLOSOne_formatting_sample_title_authors_affiliations.pdf](https://track.editorialmanager.com/CL0/https:%2F%2Fjournals.plos.org%2Fplosone%2Fs%2Ffile%3Fid=ba62%2FPLOSOne_formatting_sample_title_authors_affiliations.pdf/1/010f019d1ea72099-7699703e-35be-4396-b571-8bcffeae5500-000000/nX3n9d5fVJ8WosIiLzeiqaK2QGmXrxvx7KnivvjBWIg=255)

I have revised the manuscript to meet PLoS One’s style requirement

2. We note that the grant information you provided in the ‘Funding Information’ and ‘Financial Disclosure’ sections do not match.

When you resubmit, please ensure that you provide the complete details and the correct grant numbers for the awards you received for your study in the ‘Funding Information’ section.

I have harmonized the grant information provided in the Funding Information and Financial Disclosure sections. I have provided information regarding the grant numbers for the awards that supported this project.

3. Please note that any information related to your funding statement should not appear in any section or other areas of your manuscript. We will only publish funding information present in the Funding Statement section of the online submission form. Please remove any funding-related text from the manuscript.

All information related to funding of this study are only printed under the funding information section

4. For studies involving third-party data, we encourage authors to share any data specific to their analyses that they can legally distribute. PLOS recognizes, however, that authors may be using third-party data they do not have the rights to share. When third-party data cannot be publicly shared, authors must provide all information necessary for interested researchers to apply to gain access to the data. (<https://journals.plos.org/plosone/s/data-availability#loc-acceptable-data-access-restrictions>)

For any third-party data that the authors cannot legally distribute, they should include the following information in their Data Availability Statement upon submission:

1) A description of the data set and the third-party source
2) If applicable, verification of permission to use the data set
3) Confirmation of whether the authors received any special privileges in accessing the data that other researchers would not have
4) All necessary contact information others would need to apply to gain access to the data

The data generated by the different studies referred to in this manuscript are under the steward of the Kenya Medical Research Institute, Data Governance Committee. In the data availability statement, I included information on the protocol title and protocol numbers of the different studies. I have also clarified that all request for data need to be submitted to the committee for review and approval and have included the contact information for the committee.

*The datasets generated during and/or analysed during the current study are not publicly available because they are bound by Government of Kenya provisions, including the Data Protection Act of 2019. Data used in this analysis were sourced from the Population-based infectious disease surveillance platform (protocol numbers KEMRI SERU SCC1899 & 2761), Sanitation and antibiotic use as drivers of colonization with resistant* Escherichia coli *study (SCC 2998) and Modelling environmental suitability for resistant* Escherichia coli *carriage: An alternative approach for estimating resistance burden and informing prescriptions in developing settings (SCC 3928). All data are under the stewardship of the KEMRI’ s Data Governance Committee. De-identified data or summaries may be availed through the corresponding author upon submission of data request for consideration by the KEMRI’s Data Governance Committee. The KEMRI’s Data Governance Committee can be contacted at cghr@kemri.go.ke or telephone +254-20-22923.*

5. We notice that your supplementary table is included in the manuscript file. Please remove it and upload it with the file type 'Supporting Information'. Please ensure that each Supporting Information file has a legend listed in the manuscript after the references list.

I have moved the supplementary table into the supporting information file and have added a list of supporting information captions at the end of the manuscript under ‘supporting information’ section

**Comments to the Author**

Reviewer #1:

The title indicates that the paper has found priority effects that determine resistant *E. coli* strains in a community tested. These priority effects are not clear. The data presented are largely correlative, and using modeling. There is value in doing modeling and looking for correlations but whether any novel correlations are found here are not clear. The images of several figures are blurry, but even if they were not blurry, the text does not indicate clear or novel findings.

I thank the reviewer for these constructive comments. I have revised the manuscript to improve clarity of the findings including the ecological mechanisms inferred.

On priority effects, I have revised the text to clarify that it is the observation of signals of secondary succession, colonization legacy and positive density-dependent feedbacks that informed the priority effects inference.

Strain colonization was not just a result of direct selection (e.g., Antibiotic X selecting for Resistance X). Instead, certain antibiotics acted as disturbance that triggers reassembly processes (secondary succession signal). The models indicated community structure’s association with baseline strains abundance (colonization legacy) and high-abundance resident strains exhibited positive density-dependent feedback.

I hypothesize that when a disturbance (like cloxacillin exposure) occurs, strains, abundant in the regional pool, are better positioned to recolonize the disturbed patch and once in moves the community to a state in which they are dominant. This sequence, where the initial abundance, influence who gets to colonize and thus determine eventual community structure of disturbed patch, is what underlie the proposal of priority effects.

I agree with the reviewer that the findings are correlative and have revised the manuscript to explicitly state that these relationships are correlative. I acknowledge that the best approach to identify priority effects will be through controlled experiments which I hope to implement in future research.

Regarding figure quality, I apologize for the resolution challenges encountered during the review. The figures were checked against journal specifications using the submission software; however, it appears the PDF conversion process may have impacted clarity. I have re-uploaded all figures at a higher DPI in the hope they will meet the expected quality.

Some questions I had were:

1. Were the people who fecal samples were taken from asked about their antibiotic usage? If so, a figure connecting the antibiotic use and resistance found would have been useful.

Information on recent antibiotic exposure was collected by the study which we have cited in the methods section (Omulo *et al*, 2021, https://doi.org/10.1186/s13756-021-00886-y). Study participants reported antibiotics use in a household within a 14-day window prior household visit. Surveillance officers use visual cues to minimize recall bias. However, this data fell short in the level of resolution that was available with the PBIDS data (antibiotic type, dosage and duration of use), only reporting use or no use of antibiotic.

While adding this information into the models could have been valuable, the observation that different antibiotic results in distinct microbial community responses, suggests that such high-level information would have complicated interpretation of results. Further, in the context of colonizing resistant bacteria, we have previously shown (Ng’eno *et al* 2024, https://doi.org/10.1371/journal.pone.0302521) exposure data adjusted for time and spatial proximity as a better metric in exposure-resistant carriage relationship analyses in community settings. Information on reported antibiotic exposure would need to be integrated in the broader temporal and spatial context for detection of potential exposure-resistance carriage signals in highly connected settings like this study area.

2. Did the authors collect the fecal samples and do the experiments to detect antibiotic resistance or did they get the data from PBIDS and do modeling with the data?

Data on susceptibility patterns were obtained from Omulo *at al* 2021. The study was implemented on the PBIDS platform. It followed ~200 households sampled at random from each locality across the study area for six months (31 August 2015, to 22 January 2016). Stool samples (two per household; a child < 5 and an adult ≥ 18 years old) were collected every after 2 weeks, tested for resistance and loads of resistant *E. coli* present in a gram of stool quantified. We have cited the study in the methods section and provided a brief description of methods used for testing of resistant bacteria. I also used data on water, sanitation and hygiene practices of the households tested that was sourced from this study. Data from PBIDS (antibiotic prescription, household geolocations and person-time-of-observation) were used to characterize the exposure landscapes. Other environmental risk factors (the proximity and population density layers) were generated as part of Ng’eno *et al* 2024.

3. Was all of the data from PBIDS?

I have clarified in the methods section that part of the data used were obtained from the PBIDS platform while the other was sourced from studies implement on the platform. I have cited and provide brief description of how the data were collected in the methods section

*Cleaned draft, lines 101-102; We used data from a population-based infectious diseases surveillance (PBIDS) platform and from previous studies implemented on the same platform..*

4. Did the authors do the experiments described in lines 210 to 218 on page 10 or is it from PBIDS?

The experiments described in lines 210 to 218 were performed as part of the longitudinal (six months) study on resistance colonization among study participants in the PBIDS platform (Omulo *et al*, 2021). This study was nested on the PBIDS platform. We have cited the study and referenced the study protocol number (SSC protocol numbers 2998) in the manuscript.

*Lines 192-194; We used data on resistant* E. coli *colonization in asymptomatic individuals from a previous longitudinal study conducted in the same PBIDS population. Sample collection and processing methods have been described elsewhere...*

5. The text regarding Fig 2 does not fully explain the data shown. The correlation between Streptomycin resistant with Sulfamethoxazole, tetracycline, and trimethoprim is not mentioned.

In the Results section, I describe positive correlations among strains resistant to ampicillin, streptomycin, sulfamethoxazole, tetracycline, and trimethoprim, noting that these strains also exhibit some degree of negative correlation with the other resistant strains. I acknowledge that the caption accompanying Figure 2 may not be adequate and have added a line clarifying what the size of the bubbles represent.

*Lines 766 -767; Fig 2: ... Yellow color indicates positive correlation while blue negative correlation among the strains. The bubble size represents the strength of the correlation...*

6. Fig 3 – several of the graphs here are described as bimodal. It is not clear how the line on the graph is made and what data it is representing.

I agree that the line overlaid on the histograms could be interpreted as representing a continuous distribution of the site frequencies across varying proportions of colonizing resistant bacterial strains. Since the frequency bins are categorical rather than continuous, this presentation may be misleading. I have revised the figure to retain only the histogram displaying the frequency distribution of sites with varying relative proportions (abundance) of resistant *Escherichia coli*. The overlaid line has been removed. The black points and associated error bars represent the median and the 95% confidence intervals of the number of sites expected by chance for each abundance category. We have clarified this interpretation in the figure caption.

*Lines 772-774; The black dots represent the median, while the error bars indicate the 2.5th and 97.5th quantiles of the distribution obtained through random subsampling (n = 1000) in the null model simulations.*

7. The relevance of Fig 4 is not clear.

I have reworded the methods section to clarify that I used area under the receiver operating characteristic curve to assess classification accuracy of the overall model (model predicting occurrence distribution of all strains simultaneously) and strain specific model (model predicting occurrence distribution of each strain individually). I have also reworded the figure caption to reflect the two assessed metrics.

*Line 282-286; We examined the explanatory power using strain-specific coefficients of discrimination (Tjur’s R2) and evaluated model classification performance using the area under the receiver operating characteristic curve (AUC). Models were evaluated for their ability to predict the occurrence distribution of all strains simultaneously as well as each strain individually.*

*Lines 779-783; Fig 4: Model classification and explanatory performance fit as measured by area under the receiver operating characteristic curve AUC and Tjur R². Each point per plot represents a resistant strain while corresponding x-axis shows the strain’s abundance estimates. Left panel represents AUC estimates while right panel estimates Tjur R2 for each strain...*

8. In the covariate data from Fig 5, in what context are all of the antibiotics listed on the x-axis? From this figure, relevant covariates are not clear.

The quadratic forms of some the variables were generated in order to capture potential quadratic relationship between the variables and carriage. I have reworded the methods section to clarify this aim. I have also clarified in the figure caption that antibiotic names followed by “.2” referred to those transformed to the quadratic forms. Finally, I have revised variables group names as used in the caption for consistency.

*Lines 233-235; To account for potential quadratic relationships, we also generated quadratic forms of antibiotic exposure and weather variables.*

*Lines 786-793: Fig 5: Correlates of* Escherichia coli *strains occurrence. Figure showing correlations among dispersal-related factors; selection factors; baseline strains density; weather; age; and the occurrence of resistant* Escherichia coli *strains. Quadratic terms for antibiotic exposure variables are denoted by adding “.2” to the antibiotic name...*

9. Fig 6, the relevance of the variance proportion is not clear. The most variance is attributed to the selective section. What is the selective section referring to? What is included in there?

I agree that the variable categories underlying Figure 6 were not sufficiently described. In the original manuscript, the definition of the “selective” category and other variable groupings was unclear, and category names were used inconsistently across sections. I have added a table in the Methods section that lists all variables included in the analysis and their corresponding categories. I have also harmonized the use of category names throughout the manuscript to ensure consistency between the text, figures, and captions

*Lines 293-294; Relative proportions of variance explained were estimated both by grouped categories and by individual risk factors (Table 1).*

*Line 756; Table 1. Variables and their corresponding categories used in this analysis*

*Lines 796-801;* *Fig. 6. Proportion of total variance explained by predictor categories. The relative contribution of each predictor group to the total variance explained (see Fig. 4, right panel) is shown for individual strains. Predictor groups include dispersal‑related factors (dispersal), selective factors related to antibiotic exposure (antibiotic), baseline strain abundance (density), age, weather‑related factors (weather), and spatially structured random effects (random ID). Bar colors indicate the proportion of variance explained by each predictor category for each strain.*

10. The relevance of Figure 7 is not clear. What value does a residual joint species distribution model provide? It is not clear in the text or the figure legend.

I have provided additional text to clarify this step of the analysis. The analysis aimed at assessing effect of baseline resistant strain abundance in observed grouping (structure) of resistant strains. Two models were fitted one including all variables and the other excluding variables estimating baseline abundance of different strains across localities. Models accounting for baseline information (used all variables) eliminated the grouping pattern in residual correlation while model excluding the baseline abundance information showed distinct grouping of strains in residual correlation.

*Lines 295-300: We also assessed residual correlation patterns in models incorporating and excluding background resistant strains abundance estimates, to separate effects of the biotic environment. Specifically, we assessed the extent to which observed correlation among the strains could be explained by initial distribution patterns of strains’ abundance in the area. The models were fitted as described and only varied in consideration of the baseline strains abundance estimates*

11. Limitations of the study are discussed clearly in the discussion but the relevance to the literature of the study findings are not clear.

I have added a line summarizing potential impact of the highlighted study limitations. That it hinders generalizability of the observed findings hence need for studies that will include broader geographic scope and ecological contexts.

*Lines 493-494; These limitations make it difficult to apply our findings more broadly. Expanding geographic scope and ecological context in future work could help clarify these relationships.*

Reviewer #2:

The manuscript presents an insightful and methodologically sophisticated investigation into the ecological determinants of colonizing antibiotic-resistant *Escherichia coli* strain assemblages in a community setting. The integration of antibiotic exposure, dispersal processes, and inferred biotic interactions within a joint species distribution modeling framework is commendable and reflects a contemporary, systems-level approach to understanding antimicrobial resistance dynamics.
The study is technically sound, with appropriate use of longitudinal surveillance data, spatially explicit predictors, and Bayesian modeling techniques. The application of the HMSC framework, coupled with convergence diagnostics and complementary analyses (e.g., SparCC correlations and null model simulations), demonstrates a high level of analytical rigor. The findings regarding the roles of antibiotic selection, colonization legacy (priority effects), and dispersal are generally well supported by the data and provide valuable contributions to the field.
However, several aspects warrant careful consideration and clarification:

I would like to thank the reviewer for their generous and insightful assessment of this work. I am pleased that our approach, assessing multiple strains concurrently, resonated with the reviewer. It is also encouraging to receive such positive feedback on the analytical rigor and the ecological framing of the study. I have revised the manuscript to enhance clarity of the findings and their interpretations.

1. Interpretation of Biotic Interactions:
While the manuscript attributes observed patterns to biotic interactions and positive feedback mechanisms, these inferences are largely indirect, based on residual correlations, bimodality, and background density effects. The authors are encouraged to temper causal language and more explicitly acknowledge the inferential nature of these conclusions.

I have revised the manuscript for clarity and to emphasise that observations and interpretations made are correlative and inferential, not causative.

2. Explained Variance and Model Limitations:
The model explains a relatively modest proportion of variance (~10%), suggesting that important unmeasured factors may influence strain assemblages. This limitation should be more prominently discussed, particularly in relation to the strength and generalizability of the conclusions.

In the HMSC framework (Ovaskainen, O. and Abrego, N. (2020) Joint Species Distribution Modelling: With Applications in R. Ecology Biodiversity and Conservation. Cambridge University Press, Cambridge.), model performance is primarily assessed via species-specific *R^2^* values. While a community-level measure can be derived by averaging these values, the resulting mean is often low. This is because many communities often contain a high proportion of rare or specialist species that are inherently difficult to predict (see Fig.4, Right panel). These rare species weigh down the community average even if dominant species are modelled accurately. Such low *R^2^* values are therefore common especially in microbial community studies implemented over small spatial scale that is highly connected.

Even so, the model predictions of our species were low across each species suggesting role of other factors that carried no spatial signature. I have highlighted this as a challenge in the discussion. I have also added it as a limitation to the generalizability of the observed associations

*Clean draft, lines 470-478: This pattern suggests the influence of additional, unmeasured factors that lack a spatial signature, given that our analysis already considered spatially structured unmeasured variables through the random effect variable. We attribute this clustering, at least in part, to potential evolutionary differences between resistant and susceptible strains. Specifically, the grouping likely reflects evolutionary relatedness in genetic mechanisms underlying global resistance traits such as efflux pumping and porin modulations among the resistant E. coli strains [83]. Variations in host resources and space availability which were not considered in this study, may also affect assemblage patterns.*

*Lines 493-494; These limitations make it difficult to apply our findings more broadly. Expanding geographic scope and ecological context in future work could help clarify these relationships.*

3. Outcome Transformation:
The conversion of resistance proportions into binary presence–absence variables may result in loss of information. The authors may wish to justify this choice more explicitly or discuss its potential implications for sensitivity and interpretation.

I agree that binarization of continuous outcomes involves some trade-offs. The choice on what can be traded depends on the objective of a study. Studies aiming at identifying associations between predictors variables and occurrence (presence) of a particular outcome benefits from clear classifications of instances of occurrence and no occurrence while those aiming to assess relationship between predictor variables and prevalence/abundance of an outcome benefit from use of continuous response variable. This study set out to assess the prior; characterizing factors associated with occurrence of colonizing antibiotic-resistant *Escherichia coli* strains configurations - rather than their relative abundances. In addition, while much effort was put into quantifying the loads of the different strains, precise quantification of strain loads can be subject biological and technical variability. Factors such as stool consistency, host physiology, and recent dietary intake can influence the concentration of bacteria in a single gram of faeces. Such “noise” makes binarization a go to option for such analysis.

I have added a sentence to clarify our choice to binarize the response variables.

*Lines 221-223: Since we set out to evaluate association between predictor variables and occurrence of colonizing antibiotic resistant* E. coli *strain assemblages (rather than their relative abundance)*

4. Spatial Interpolation of Predictors:
The reliance on interpolated antibiotic exposure and background density surfaces introduces potential spatial smoothing biases. A brief discussion of the uncertainty associated with these interpolations would strengthen the methodological transparency.

I appreciate the reviewer’s comment regarding the potential for spatial smoothing biases. The decision to use spatiotemporally summarized estimates rather than individual-level data was intentional, as these metrics are more likely to accurately reflect the spatiotemporal scales at which ecological and evolutionary processes which modulate carriage emergence and distribution occur (<https://doi.org/10.1371/journal.pone.0302521>).

Beyond the individual traits, resistance is also an emergent property of a microbial community. Interactions within community members can determine response and evolutionary trajectory of resistant strains. These community assembles by movements of bacteria between connecting metacommunities. Thus, antibiotic consumed next door, several weeks or months ago needs to be accounted for when generating exposure estimates.

The raster data layers used summarized antibiotic exposure information from every household in the study area (between 2010 and 2015) for a truer reflection of the exposure landscape. Unlike individual-level metrics, these raster layers capture historical and spatial dependencies necessary to assess colonization risk in a community setting.

To minimize effects of data holes and other spatial smoothing biases, the layers generated used point data for every household present in the study area during the period 2010 and 2015.

I have reworded the line on interpolation of household-level antibiotic exposure estimates to clarify this.

*Lines 156- 157: We extended the risk estimates across space by interpolating among all PBIDS households’ exposure point data using the cubic spline method in QGIS (version 3.22.1)*

5. Data Availability:
The data are not fully publicly available and are accessible only upon request and approval due to regulatory constraints. While this is understandable, it limits reproducibility and should be clearly aligned with the journal’s data-sharing policies.

I acknowledge the reviewer’s comment regarding the importance of data accessibility for reproducibility. Due to legal and ethical constraints governed by the Kenya Medical Research Institute (KEMRI), the raw data cannot be made fully public. However, in the interest of open science, I have revised the data availability statement to clearly outline the specific conditions under which the data can be shared. I have also provided the contact information for the KEMRI Data Governance Committee which serve as the steward for this data. They are responsible for processing data requests in accordance with institutional sharing policies. I will also consult with the journal’s editorial office to ensure our statement fully aligns with their specific requirements for restricted datasets.

6. Language and Presentation:
The manuscript is generally well written and intelligible; however, minor grammatical inconsistencies, occasional awkward phrasing, and overly long sentences are present. A thorough language edit would improve clarity and readability.

I thank the reviewer for the thorough assessment of this manuscript, I have revised the language to address grammatical inconsistencies, refine sentence structure, and improve clarity and readability.

7. Scope and Generalizability:
The study is conducted within a single, highly specific setting. The authors should further emphasize the context-dependent nature of their findings and exercise caution in extrapolating to broader populations.

I acknowledge the limitation with regards to generalization of the observed findings. I have added a statement indicating that the limited scope of the study binds the ability to generalize these finding over all settings and that studies with broader spatial and ecological reach will be needed. I have also revised the title of the study to clarify that the findings are in relation to the setting studied.

*Lines 1-3: Antibiotics and priority effects shape colonizing resistant* Escherichia coli *strain assemblages in a community setting*

*Lines 495-498; These limitations along with the overall limited scope of the study make it difficult to apply our findings more broadly. Expanding geographic scope and ecological context in future work could help clarify these relationships.*
