# Supplementary material for: Antibiotics and priority effects on colonizing resistant Escherichia coli strain assemblages in a community setting
Source: PLoS One. 2026 Jun 24;21(6):e0352163. doi: 10.1371/journal.pone.0352163 (PMC13293415; doi:10.1371/journal.pone.0352163)
Supplement: Peer review history

# Peer review: Decision Letter 0

**DOI:** 10.1371/journal.pone.0352163.r001

**Authors:** Eric Ogola

24 Mar 2026

PONE-D-26-03469

Antibiotics and priority effects determine colonizing resistant Escherichia coli strain assemblages in community settings

PLOS One

Dear Dr. Ng’eno,

Thank you for submitting your manuscript to PLOS ONE. After careful consideration, we feel that it has merit but does not fully meet PLOS ONE’s publication criteria as it currently stands. Therefore, we invite you to submit a revised version of the manuscript that addresses the points raised during the review process.

Kindly address the comments from the two reviewers.

Please submit your revised manuscript by May 08 2026 11:59PM. If you will need more time than this to complete your revisions, please reply to this message or contact the journal office at plosone@plos.org. When you're ready to submit your revision, log on to https://www.editorialmanager.com/pone/ and select the ’submissions Needing Revision' folder to locate your manuscript file.

Please include the following items when submitting your revised manuscript:

- A letter that responds to each point raised by the academic editor and reviewer(s). You should upload this letter as a separate file labeled 'Response to Reviewers'.
- A marked-up copy of your manuscript that highlights changes made to the original version. You should upload this as a separate file labeled 'Revised Manuscript with Track Changes'.
- An unmarked version of your revised paper without tracked changes. You should upload this as a separate file labeled 'Manuscript'.

If you would like to make changes to your financial disclosure, please include your updated statement in your cover letter. Guidelines for resubmitting your figure files are available below the reviewer comments at the end of this letter.

If applicable, we recommend that you deposit your laboratory protocols in protocols.io to enhance the reproducibility of your results. Protocols.io assigns your protocol its own identifier (DOI) so that it can be cited independently in the future. For instructions see: https://journals.plos.org/plosone/s/submission-guidelines#loc-laboratory-protocols. Additionally, PLOS ONE offers an option for publishing peer-reviewed Lab Protocol articles, which describe protocols hosted on protocols.io. Read more information on sharing protocols at https://plos.org/protocols?utm_medium=editorial-email&utm_source=authorletters&utm_campaign=protocols.

We look forward to receiving your revised manuscript.

Kind regards,

Eric Ogola, MPH

Academic Editor

PLOS One

Journal Requirements:

When submitting your revision, we need you to address these additional requirements.

1. Please ensure that your manuscript meets PLOS ONE’s style requirements, including those for file naming. The PLOS ONE style templates can be found at

https://journals.plos.org/plosone/s/file?id=wjVg/PLOSOne_formatting_sample_main_body.pdf and https://journals.plos.org/plosone/s/file?id=ba62/PLOSOne_formatting_sample_title_authors_affiliations.pdf

2. We note that the grant information you provided in the ‘Funding Information’ and ‘Financial Disclosure’ sections do not match.

When you resubmit, please ensure that you provide the complete details and the correct grant numbers for the awards you received for your study in the ‘Funding Information’ section.

3. Please note that any information related to your funding statement should not appear in any section or other areas of your manuscript. We will only publish funding information present in the Funding Statement section of the online submission form. Please remove any funding-related text from the manuscript.

4. For studies involving third-party data, we encourage authors to share any data specific to their analyses that they can legally distribute. PLOS recognizes, however, that authors may be using third-party data they do not have the rights to share. When third-party data cannot be publicly shared, authors must provide all information necessary for interested researchers to apply to gain access to the data. (https://journals.plos.org/plosone/s/data-availability#loc-acceptable-data-access-restrictions)

For any third-party data that the authors cannot legally distribute, they should include the following information in their Data Availability Statement upon submission:

1) A description of the data set and the third-party source

2) If applicable, verification of permission to use the data set

3) Confirmation of whether the authors received any special privileges in accessing the data that other researchers would not have

4) All necessary contact information others would need to apply to gain access to the data

5. We notice that your supplementary table is included in the manuscript file. Please remove it and upload it with the file type ’supporting Information'. Please ensure that each Supporting Information file has a legend listed in the manuscript after the references list.

If the reviewer comments include a recommendation to cite specific previously published works, please review and evaluate these publications to determine whether they are relevant and should be cited. There is no requirement to cite these works unless the editor has indicated otherwise.

[Note: HTML markup is below. Please do not edit.]

Reviewers' comments:

Reviewer’s Responses to Questions

**Comments to the Author**

1. Is the manuscript technically sound, and do the data support the conclusions?

The manuscript must describe a technically sound piece of scientific research with data that supports the conclusions. Experiments must have been conducted rigorously, with appropriate controls, replication, and sample sizes. The conclusions must be drawn appropriately based on the data presented.

Reviewer #1: No

Reviewer #2: Partly

**********

2. Has the statistical analysis been performed appropriately and rigorously?

Reviewer #1: No

Reviewer #2: Yes

**********

3. Have the authors made all data underlying the findings in their manuscript fully available?

The PLOS Data policy requires authors to make all data underlying the findings described in their manuscript fully available without restriction, with rare exception (please refer to the Data Availability Statement in the manuscript PDF file). The data should be provided as part of the manuscript or its supporting information, or deposited to a public repository. For example, in addition to summary statistics, the data points behind means, medians and variance measures should be available. If there are restrictions on publicly sharing data—e.g. participant privacy or use of data from a third party—those must be specified.

Reviewer #1: No

Reviewer #2: No

**********

4. Is the manuscript presented in an intelligible fashion and written in standard English?

PLOS ONE does not copyedit accepted manuscripts, so the language in submitted articles must be clear, correct, and unambiguous. Any typographical or grammatical errors should be corrected at revision, so please note any specific errors here.

Reviewer #1: Yes

Reviewer #2: Yes

**********

5. Review Comments to the Author

Please use the space provided to explain your answers to the questions above. You may also include additional comments for the author, including concerns about dual publication, research ethics, or publication ethics. (Please upload your review as an attachment if it exceeds 20,000 characters)

Reviewer #1: The title indicates that the paper has found priority effects that determine resistant E. coli strains in a community tested. These priority effects are not clear. The data presented are largely correlative, and using modeling. There is value in doing modeling and looking for correlations but whether any novel correlations are found here are not clear. The images of several figures are blurry, but even if they were not blurry, the text does not indicate clear or novel findings.

Some questions I had were:

1. Were the people who fecal samples were taken from asked about their antibiotic usage? If so, a figure connecting the antibiotic use and resistance found would have been useful.

2. Did the authors collect the fecal samples and do the experiments to detect antibiotic resistance or did they get the data from PBIDS and do modeling with the data?

3. Was all of the data from PBIDS?

4. Did the authors do the experiments described in lines 210 to 218 on page 10 or is it from PBIDS?

5. The text regarding Fig 2 does not fully explain the data shown. The correlation between Streptomycin resistant with Sulfamethoxazole, tetracycline, and trimethoprim is not mentioned.

6. Fig 3 – several of the graphs here are described as bimodal. It is not clear how the line on the graph is made and what data it is representing.

7. The relevance of Fig 4 is not clear.

8. In the covariate data from Fig 5, in what context are all of the antibiotics listed on the x-axis? From this figure, relevant covariates are not clear.

9. Fig 6, the relevance of the variance proportion is not clear. The most variance is attributed to the selective section. What is the selective section referring to? What is included in there?

10. The relevance of Figure 7 is not clear. What value does a residual joint species distribution model provide? It is not clear in the text or the figure legend.

Limitations of the study are discussed clearly in the discussion but the relevance to the literature of the study findings are not clear.

Reviewer #2: The manuscript presents an insightful and methodologically sophisticated investigation into the ecological determinants of colonizing antibiotic-resistant Escherichia coli strain assemblages in a community setting. The integration of antibiotic exposure, dispersal processes, and inferred biotic interactions within a joint species distribution modeling framework is commendable and reflects a contemporary, systems-level approach to understanding antimicrobial resistance dynamics.

The study is technically sound, with appropriate use of longitudinal surveillance data, spatially explicit predictors, and Bayesian modeling techniques. The application of the HMSC framework, coupled with convergence diagnostics and complementary analyses (e.g., SparCC correlations and null model simulations), demonstrates a high level of analytical rigor. The findings regarding the roles of antibiotic selection, colonization legacy (priority effects), and dispersal are generally well supported by the data and provide valuable contributions to the field.

However, several aspects warrant careful consideration and clarification:

1. Interpretation of Biotic Interactions:

While the manuscript attributes observed patterns to biotic interactions and positive feedback mechanisms, these inferences are largely indirect, based on residual correlations, bimodality, and background density effects. The authors are encouraged to temper causal language and more explicitly acknowledge the inferential nature of these conclusions.

2. Explained Variance and Model Limitations:

The model explains a relatively modest proportion of variance (~10%), suggesting that important unmeasured factors may influence strain assemblages. This limitation should be more prominently discussed, particularly in relation to the strength and generalizability of the conclusions.

3. Outcome Transformation:

The conversion of resistance proportions into binary presence–absence variables may result in loss of information. The authors may wish to justify this choice more explicitly or discuss its potential implications for sensitivity and interpretation.

4. Spatial Interpolation of Predictors:

The reliance on interpolated antibiotic exposure and background density surfaces introduces potential spatial smoothing biases. A brief discussion of the uncertainty associated with these interpolations would strengthen the methodological transparency.

5. Data Availability:

The data are not fully publicly available and are accessible only upon request and approval due to regulatory constraints. While this is understandable, it limits reproducibility and should be clearly aligned with the journal’s data-sharing policies.

6. Language and Presentation:

The manuscript is generally well written and intelligible; however, minor grammatical inconsistencies, occasional awkward phrasing, and overly long sentences are present. A thorough language edit would improve clarity and readability.

7. Scope and Generalizability:

The study is conducted within a single, highly specific setting. The authors should further emphasize the context-dependent nature of their findings and exercise caution in extrapolating to broader populations.

Despite these limitations, the manuscript addresses an important and timely topic and offers meaningful ecological insights into antimicrobial resistance dynamics. With minor revisions to improve clarity, temper interpretation, and address methodological considerations, the manuscript would be suitable for publication.

**********

6. PLOS authors have the option to publish the peer review history of their article (what does this mean?). If published, this will include your full peer review and any attached files.

If you choose “no”, your identity will remain anonymous but your review may still be made public.

**Do you want your identity to be public for this peer review?**  For information about this choice, including consent withdrawal, please see our Privacy Policy.

Reviewer #1: No

Reviewer #2: No

**********

[NOTE: If reviewer comments were submitted as an attachment file, they will be attached to this email and accessible via the submission site. Please log into your account, locate the manuscript record, and check for the action link "View Attachments". If this link does not appear, there are no attachment files.]

To ensure your figures meet our technical requirements, please review our figure guidelines: https://journals.plos.org/plosone/s/figures

You may also use PLOS’s free figure tool, NAAS, to help you prepare publication quality figures: https://journals.plos.org/plosone/s/figures#loc-tools-for-figure-preparation.

NAAS will assess whether your figures meet our technical requirements by comparing each figure against our figure specifications.

# Author comment: Author response to Decision Letter 1

**DOI:** 10.1371/journal.pone.0352163.r002

30 Apr 2026

I have attached with this submission a file with response to the reviewer’s comments

--------------------------------------------------------------------

PONE-D-26-03469R1

Antibiotics and priority effects shape colonizing resistant Escherichia coli strain assemblages in a community setting

Eric Ng’eno

Dear Dr. Ng’eno,

We've checked your submission and before we can proceed, we need you to address the following issues:

1. Please upload a copy of Figure 1 to 7 which you refer to in your text on page 33-35. Or if the figure is no longer to be included as part of the submission please remove all reference to it within the text.

>>> I have uploaded all figures 1 to 7

2. Please also provide a non-author point of contact (e.g., data access committee, ethics committee, or other institutional body) where data requests may be made. Note that it is not acceptable for the authors to be the sole named individuals responsible for ensuring data access.

>>> I did include contact information for the committee that oversites all data used in this analysis (please see below an excerpt of my data statement)

De-identified data or summaries may be availed through the corresponding author upon submission of data request for consideration by the KEMRI’s Data

Governance Committee. The KEMRI’s Data Governance Committee can be contacted at cghr@kemri.go.ke or telephone +254-20-22923.

Attachment Submitted filename: Response to Reviewers.docx

# Peer review: Decision Letter 1

**DOI:** 10.1371/journal.pone.0352163.r003

**Authors:** Eric Ogola, Muhammad Hussnain Siddique

7 Jun 2026

Antibiotics and priority effects shape colonizing resistant Escherichia coli strain assemblages in a community setting

PONE-D-26-03469R1

Dear Dr. Ng’eno,

We’re pleased to inform you that your manuscript has been judged scientifically suitable for publication and will be formally accepted for publication once it meets all outstanding technical requirements.

Within one week, you’ll receive an e-mail detailing the required amendments. When these have been addressed, you’ll receive a formal acceptance letter and your manuscript will be scheduled for publication.

An invoice will be generated when your article is formally accepted. Please note, if your institution has a publishing partnership with PLOS and your article meets the relevant criteria, all or part of your publication costs will be covered. Please make sure your user information is up-to-date by logging into Editorial Manager at Editorial Manager® and clicking the ‘Update My Information' link at the top of the page. For questions related to billing, please contact billing support.

If your institution or institutions have a press office, please notify them about your upcoming paper to help maximize its impact. If they’ll be preparing press materials, please inform our press team as soon as possible -- no later than 48 hours after receiving the formal acceptance. Your manuscript will remain under strict press embargo until 2 pm Eastern Time on the date of publication. For more information, please contact onepress@plos.org.

Kind regards,

Muhammad Hussnain Siddique

Academic Editor

PLOS One

Additional Editor Comments (optional):

Reviewers' comments:

Reviewer’s Responses to Questions

**Comments to the Author**

1. If the authors have adequately addressed your comments raised in a previous round of review and you feel that this manuscript is now acceptable for publication, you may indicate that here to bypass the “Comments to the Author” section, enter your conflict of interest statement in the “Confidential to Editor” section, and submit your "Accept" recommendation.

Reviewer #1: All comments have been addressed

Reviewer #2: All comments have been addressed

**********

2. Is the manuscript technically sound, and do the data support the conclusions?

The manuscript must describe a technically sound piece of scientific research with data that supports the conclusions. Experiments must have been conducted rigorously, with appropriate controls, replication, and sample sizes. The conclusions must be drawn appropriately based on the data presented.

Reviewer #1: Yes

Reviewer #2: Yes

**********

3. Has the statistical analysis been performed appropriately and rigorously?

Reviewer #1: Yes

Reviewer #2: Yes

**********

4. Have the authors made all data underlying the findings in their manuscript fully available?

The PLOS Data policy requires authors to make all data underlying the findings described in their manuscript fully available without restriction, with rare exception (please refer to the Data Availability Statement in the manuscript PDF file). The data should be provided as part of the manuscript or its supporting information, or deposited to a public repository. For example, in addition to summary statistics, the data points behind means, medians and variance measures should be available. If there are restrictions on publicly sharing data—e.g. participant privacy or use of data from a third party—those must be specified.

Reviewer #1: Yes

Reviewer #2: Yes

**********

5. Is the manuscript presented in an intelligible fashion and written in standard English?

PLOS ONE does not copyedit accepted manuscripts, so the language in submitted articles must be clear, correct, and unambiguous. Any typographical or grammatical errors should be corrected at revision, so please note any specific errors here.

Reviewer #1: Yes

Reviewer #2: Yes

**********

6. Review Comments to the Author

Please use the space provided to explain your answers to the questions above. You may also include additional comments for the author, including concerns about dual publication, research ethics, or publication ethics. (Please upload your review as an attachment if it exceeds 20,000 characters)

Reviewer #1: (No Response)

Reviewer #2: The manuscript entitled “Antibiotics and priority effects shape colonizing resistant Escherichia coli strain assemblages in a community setting” addresses an important and timely topic concerning antimicrobial resistance ecology in densely populated community settings. The study integrates ecological theory with epidemiological analysis and provides novel insights into the role of antibiotic exposure, dispersal, and priority effects in shaping resistant E. coli assemblages. The manuscript is generally well structured, scientifically relevant, and supported by an extensive dataset. All the concerns were addressed satisfactorily.

**********

7. PLOS authors have the option to publish the peer review history of their article (what does this mean?). If published, this will include your full peer review and any attached files.

If you choose “no”, your identity will remain anonymous but your review may still be made public.

**Do you want your identity to be public for this peer review?**  For information about this choice, including consent withdrawal, please see our Privacy Policy.

Reviewer #1: No

Reviewer #2: No

**********

# Editor report: Acceptance letter

**DOI:** 10.1371/journal.pone.0352163.r004

**Authors:** Eric Ogola, Muhammad Hussnain Siddique

PONE-D-26-03469R1

PLOS One

Dear Dr. Ng’eno,

I'm pleased to inform you that your manuscript has been deemed suitable for publication in PLOS One. Congratulations! Your manuscript is now being handed over to our production team.

At this stage, our production department will prepare your paper for publication. This includes ensuring the following:

* All references, tables, and figures are properly cited

* All relevant supporting information is included in the manuscript submission,

* There are no issues that prevent the paper from being properly typeset

You will receive further instructions from the production team, including instructions on how to review your proof when it is ready. Please keep in mind that we are working through a large volume of accepted articles, so please give us a few days to review your paper and let you know the next and final steps.

Lastly, if your institution or institutions have a press office, please let them know about your upcoming paper now to help maximize its impact. If they'll be preparing press materials, please inform our press team within the next 48 hours. Your manuscript will remain under strict press embargo until 2 pm Eastern Time on the date of publication. For more information, please contact onepress@plos.org.

You will receive an invoice from PLOS for your publication fee after your manuscript has reached the completed accept phase. If you receive an email requesting payment before acceptance or for any other service, this may be a phishing scheme. Learn how to identify phishing emails and protect your accounts at https://explore.plos.org/phishing.

If we can help with anything else, please email us at customercare@plos.org.

Thank you for submitting your work to PLOS ONE and supporting open access.

Kind regards,

PLOS ONE Editorial Office Staff

on behalf of

Dr. Muhammad Hussnain Siddique

Academic Editor

PLOS One
